# Heterogeneity analysis of the *CEBPA*dm AML based on bZIP region mutations

**DOI:** 10.1097/BS9.0000000000000153

**Published:** 2023-03-06

**Authors:** Yan Hui, Shuxin Li, Junping Zhang, Bingcheng Liu, Yingchang Mi, Hui Wei, Jianxiang Wang

**Affiliations:** a State Key Laboratory of Experimental Hematology, National Clinical Research Center for Blood Diseases, Haihe Laboratory of Cell Ecosystem, Institute of Hematology & Blood Diseases Hospital, Chinese Academy of Medical Sciences & Peking Union Medical College, Tianjin 300020, China; b Tianjin Institutes of Health Science, Tianjin 301600, China

**Keywords:** Acute myeloid leukemia, *CEBPA* mutations, Heterogeneity, Outcome

## Abstract

Patients with double-mutated *CEBPA* (*CEBPA*dm) AML were stratified into favorable risk group, however, few studies have investigated the heterogeneity of different *CEBPA*dm types in detail. In this study, we analyzed 2211 newly diagnosed AML and identified *CEBPA*dm in 10.8% of the patients. Within the *CEBPA*dm cohort, 225 of 239 patients (94.14%) presented with bZIP region mutations (*CEBPA*dmbZIP) while 14 of 239 patients (5.86%) without bZIP region mutation (*CEBPA*dmnonbZIP). Analysis of the accompanied molecular mutations showed statistically different incidences of GATA2 mutations between the *CEBPA*dmbZIP group and the *CEBPA*dmnonbZIP group (30.29% vs 0%). In the analysis of outcomes, patients with *CEBPA*dmnonbZIP were associated with shorter overall survival (OS) censored at hematopoietic stem cell transplantation (HSCT) during CR1 compared to those with *CEBPA*dmbZIP (hazard ratio (HR) = 3.132, 95% confidence interval (CI) = 1.229–7.979, *P* = .017). Refractory or relapsed AML (R/RAML) patients with *CEBPA*dmnonbZIP were associated with shorter OS compared to those with *CEBPA*dmbZIP (HR = 2.881, 95% CI = 1.021–8.131, *P* = .046). Taken together, AML with *CEBPA*dmbZIP and *CEBPA*dmnonbZIP showed different outcomes and might be regarded as distinctive AML entities.

## 1. INTRODUCTION

The mutations of the gene encoding the CCAAT-enhancer binding protein alpha (CEBPA) were found in 6% to 15% of newly-diagnosed Acute Myeloid Leukemia (AML) patients, two-thirds of which were double-mutated *CEBPA* (*CEBPA*dm).^1–3^ Initially, *CEBPA*dm was stratified into favorable risk group.^4–7^ Recently, studies demonstrated that the presence of inframe bZIP mutations of *CEBPA* was associated with favorable prognosis in newly diagnosed AML^2,8,9^ which was updated in the new International Consensus Classification (ICC) of Myeloid Neoplasms and Acute Leukemia.^10^ However, *CEBPA*dm without bZIP region mutation (*CEBPA*dmnonbZIP) is classified as CEBPA mutated AML in the 5th edition of WHO Classification of Haematolymphoid Tumours^11^ but not in ICC classification. Few studies have investigated the impact of different *CEBPA*dm types in detail, especially for *CEBPA*dmnonbZIP. To further evaluate the heterogeneity of *CEBPA*dm, we analyzed the clinical features and outcomes of patients with different mutation types of *CEBPA*dm.

## 2. METHODS

### 2.1. Patients

Two hundred and thirty-nine newly diagnosed AML patients with *CEBPA*dm from 2211 consecutive AML (non-APL) patients treated in our center between July 2011 and December 2021 were assessed for the inclusion criteria including the following: AML according to WHO classification (version 2016)^12^; Patients received at least 2 courses of chemotherapy treatment if patients did not achieve complete response (CR) after the first course while others at least 1 course. The study was approved by the hospital ethics committee (approval number: NSFC2021076-EC-2) and conducted in accordance with the Declaration of Helsinki.

### 2.2. Genetic studies

Genetic analysis was performed on bone marrow samples obtained from patients at initial presentation. Cytogenetics were analyzed by R-banding, and the molecular genetic examination was performed by next-generation sequencing (NGS) and polymerase chain reaction (PCR).

### 2.3. Statistical analysis

Event-free survival (EFS) was defined as the interval from diagnosis to assessment of response after the second course of chemotherapy treatment if patients failed to achieve CR in the first induction chemotherapy, the date of relapse, or the date of death, whichever occurred first. Overall survival (OS) was calculated from the date of AML diagnosis to the date of death or the date of last follow-up for surviving patients. To exclude the bias brought about by early relapse or death ahead of hematopoietic stem cell transplantation (HSCT) during the first complete remission phase (CR1), landmark analysis were used. For considering the impact of HSCT on OS, the landmark was set on the median time from trial-enrollment to CR1-HSCT. Statistical analysis were performed using SPSS (version 21.0) and GraphPad Prism (version 7.0) software. Univariable analysis were using the Cox proportional hazards regression model. Statistical tests were 2-sided with a significance level set at 0.05.

## 3. RESULTS

### 3.1. Patients

**Figure 1.**
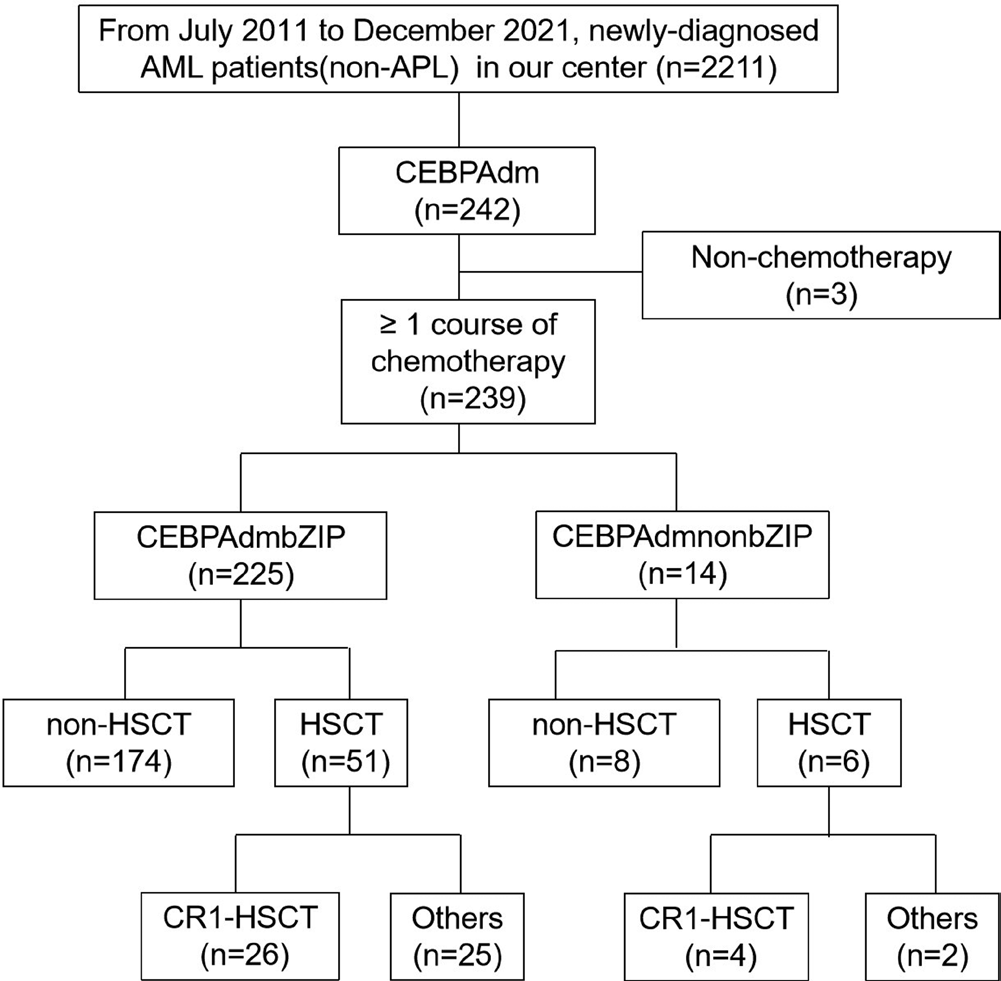
Flow diagram of patients selection. AML = Acute Myeloid Leukemia, APL = Acute Promyelocytic Leukemia, CEBPAdm = double-mutated CEBPA, CEBPAdmbZIP = mutations occurred in bZIP region, CEBPAdmnonbZIP = mutations did not occur in bZIP region, HSCT = hematopoietic stem cell transplantation.

**Figure 2.**
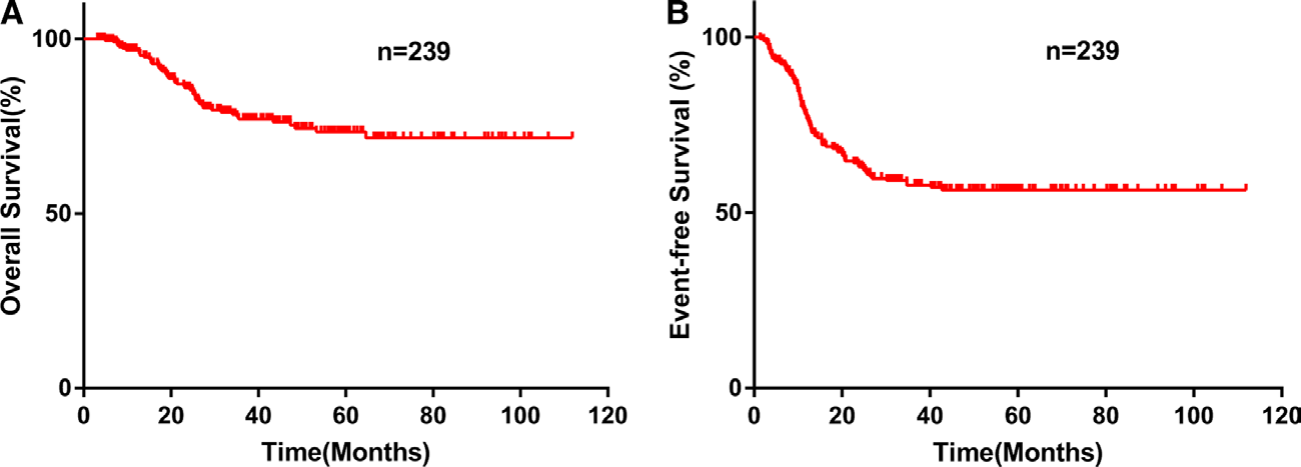
The entire cohort outcomes of CEBPAdm AML: (A) OS, (B) EFS. CEBPAdm = double-mutated *CEBPA*, EFS = event-free survival, OS = overall survival.

Totally, 2211 newly-diagnosed AML (non-APL) patients in our center were screened and 242 patients with *CEBPA*dm were found. Two hundred thirty-nine patients with *CEBPA*dm were included in the study while 3 patients who did not receive chemotherapy were excluded (Figure 1). The baseline data of the 239 AML patients with *CEBPA*dm are shown in Supplementary Table 1, http://links.lww.com/BS/A55. Of these patients, the median age was 40 years (interquartile range (IQR) = 30–48 years) and the median follow-up period was 42.43 months (IQR = 19.57–60.57 months). In our cohort, 7 + 3 induction regimens which consists of DA (Daunorubicin, Cytarabine) or IA (Idarubicin, Cytarabine) were used as the first induction therapy in 154 patients (64.44%), while HAD (homoharringtonine, cytarabine, daunorubicin)^13,14^ was used in 76 patients (31.80%). Thirty patients underwent HSCT during CR1. The median OS and EFS were 33.53 months (IQR = 17.20–59.13 months) and 23.70 months (IQR = 10.30–56.00 months), respectively (the Kaplan-Meier curves are shown in Figure 2).

### 3.2. Comparison of clinical features between *CEBPA*dmbZIP and *CEBPA*dmnonbZIP

Based on the location of mutations, *CEBPA*dm was divided into 2 types: At least 1 of the 2 mutations occurred in bZIP region (*CEBPA*dmbZIP); Mutations did not occur in bZIP region (*CEBPA*dmnonbZIP). In our cohort, *CEBPA*dmbZIP was found in 225 of 239 patients (94.14%) while 14 of 239 patients (5.86%) presented with *CEBPA*dmnonbZIP. We compared the clinical features of the 2 groups and focused on the accompanied molecular mutations. Table 1 shows that baseline characteristics were balanced among the 2 groups which included age, sex, white blood cell count (WBC), hemoglobin (HB) content, platelet count (PLT), and cytogenetics (*P* > .05). Intriguingly, *CEBPA*dmnonbZIP patients had no *GATA2* mutation at all, while 53 of 175 (30.29%) *CEBPA*dm bZIP group patients happened (*P* = .034). There was no statistical difference in the rest of the molecular markers and treatments between the 2 groups. The CR rate after induction chemotherapy and the measurable residual disease (MRD)-positive rate of CEBPAdmbZIP group and CEBPAdmnonbZIP group presented no statistical difference (CR rate: 90.67% vs 85.71%, *P* = .887; MRD-positive rate: 11.58% vs 8.33%, *P* = 1.000).

**Table 1:** Comparison of clinical characteristics, karyotypes, genetic molecular abnormalities, and treatments between CEBPAdmbZIP and CEBPAdmnonbZIP.

	CEBPAdmbZIP	CEBPAdmnonbZIP	*P* value
n	225	14	
Age, y median (IQR)	40.00 (29.00–48.00)	39.00 (33.75–50.75)	.398
Sex			.170
Male	138	6	
Female	87	8	
Laboratory, median (IQR)			
WBC, ×10^9^/L	20.05 (8.00–54.14)	17.90 (5.82–57.19)	.605
HB, ×g/L	96.00 (80.00–114.50)	80.50 (76.50–95.00)	.305
PLT, ×10^9^/L	31.00 (20.00–49.00)	37.00 (29.75–82.50)	.055
Karyotypes			.081
Normal	176	8	
Abnormal	42	6	
Unknown	7	0	
Molecular markers			
*FLT3-ITD* (n = 239)	16/225	1/14	1.000
*FLT3-TKD* (n = 239)	7/225	0/14	1.000
*CSF3R* (n = 189)	18/175	2/14	.987
*GATA2* (n = 189)	53/175	0/14	.034
*NPM1* (n = 189)	4/175	1/14	.822
*WT1* (n = 189)	31/175	1/14	.519
*TET2* (n = 189)	16/175	2/14	.528
*NRAS* (n = 189)	30/175	2/14	1.00
*ASXL1* (n = 189)	4/175	1/14	.822
*IDH1/2* (n = 189)	4/175	0/14	1.000
Induction chemotherapy			.485
HAD	70	6	
DA	125	8	
IA	21	0	
Others	9	0	
CR1-HSCT	26/225	4/14	.062
R/RAML	80	5	1.000

The bold *P* value indicates statistical significance with *P* less than 0.05.

CR1-HSCT = HSCT during the first complete remission phase, DA = daunorubicin and cytarabine, HAD = homoharringtonine, cytarabine, and daunorubicin, HB = hemoglobin, IA = idarubicin and cytarabine, IQR = interquartile range; PLT = platelet, R/RAML = refractory or relapsed AML, WBC = white blood cell.

### 3.3. Comparison of outcomes between *CEBPA*dmbZIP and *CEBPA*dmnonbZIP

**Figure 3.**
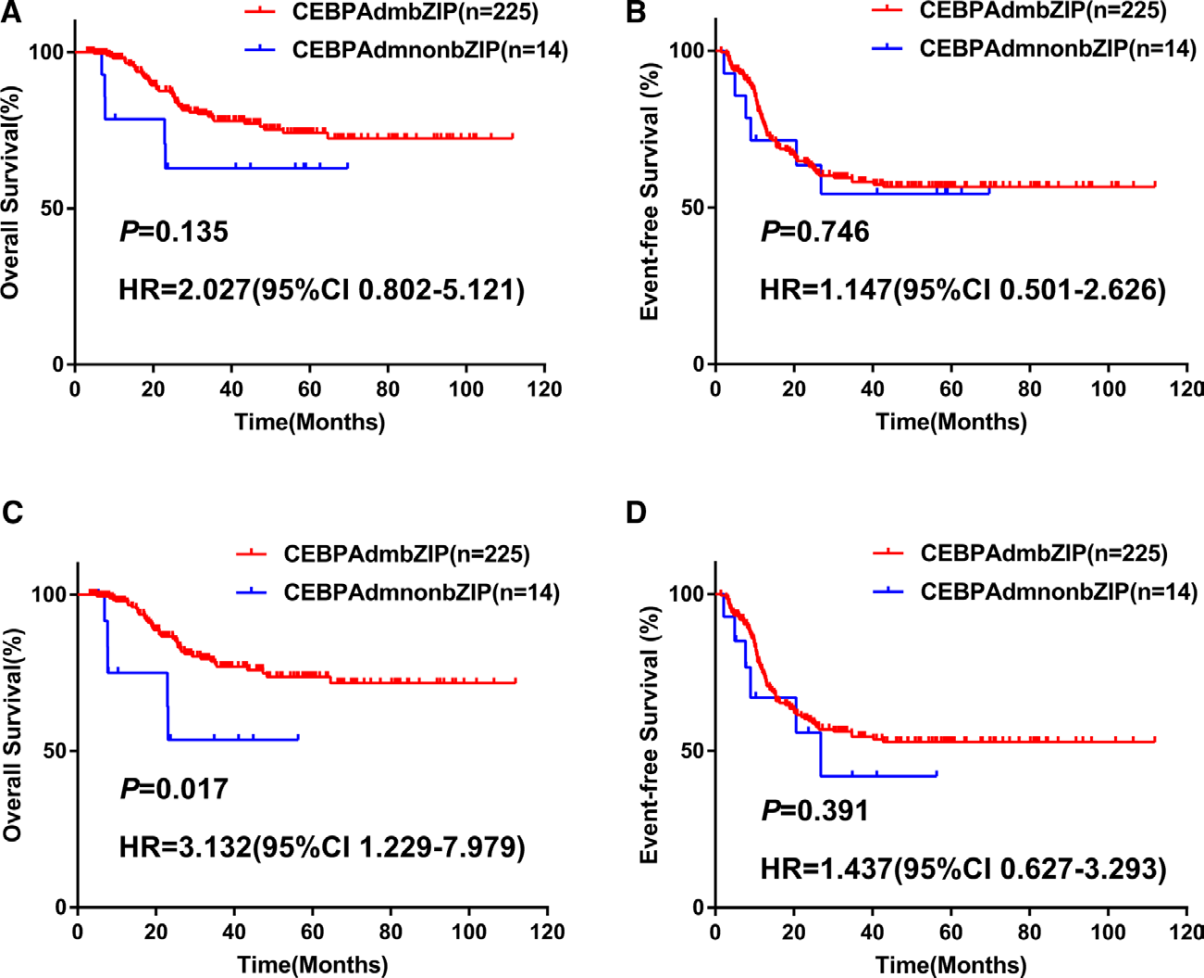
Survival analysis according to CEBPAdm types. OS and EFS of CEBPAdmbZIP and CEBPAdmnonbZIP AML (A and B), OS and EFS censored at HSCT during CR1 of CEBPAdmbZIP and CEBPAdmnonbZIP AML (C and D). AML = Acute Myeloid Leukemia, HR = hazard ratio, CI = confidence interval, CEBPAdm = double-mutated *CEBPA*, CEBPAdmnonbZIP = mutations did not occur in bZIP region, EFS = event-free survival, HSCT = hematopoietic stem cell transplantation, OS = overall survival.

**Figure 4.**
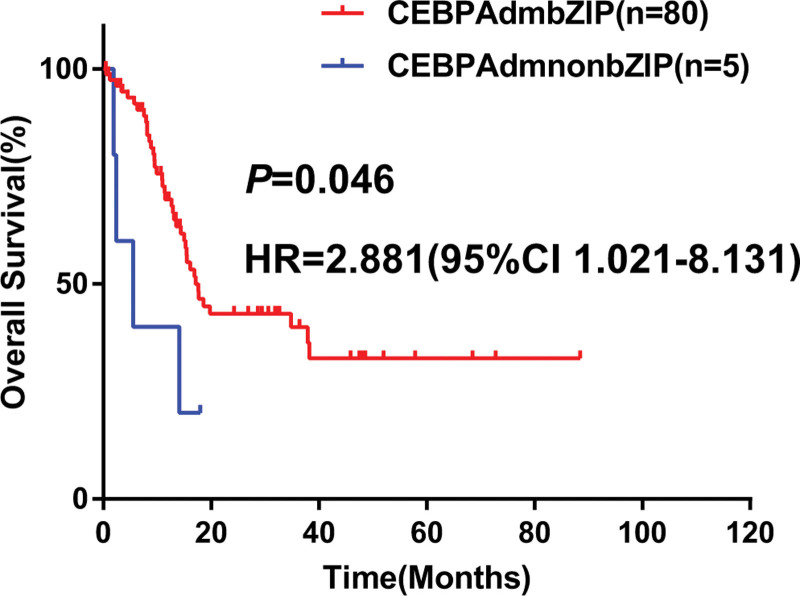
Survival analysis of R/RAML according to CEBPAdm types. AML = Acute Myeloid Leukemia, CEBPAdm = double-mutated *CEBPA*, CEBPAdmnonbZIP = mutations did not occur in bZIP region, R/RAML = refractory or relapsed AML.

Further, we investigated the impact of *CEBPA*dm type on outcomes. In univariate analysis by the Cox proportional hazards regression model, OS and EFS present no statistical difference (OS: hazard ratio (HR) = 2.027, 95% confidence interval (CI) = 0.802–5.121, *P* = .135; EFS: HR = 1.147, 95% CI = 0.501–2.626, *P* = .746, the Kaplan-Meier curves are shown in Figure 3A, B). When HSCT during CR1 was considered as a censored event, patients with *CEBPA*dmnonbZIP were associated with shorter OS censored at HSCT compared to those with *CEBPA*dmbZIP (HR = 3.132, 95% CI = 1.229–7.979, *P* = .017, the Kaplan-Meier curves are shown in Figure 3C). However, no statistical difference was found in EFS censored at HSCT between *CEBPA*dmnonbZIP and *CEBPA*dmbZIP (HR = 1.437, 95% CI = 0.627–3.293, *P* = .391, the Kaplan-Meier curves are shown in Figure 3D). The discordant results for OS and EFS remind us to focus on outcomes of patients with refractory or relapsed AML (R/RAML). Eighty-five patients were R/RAML in the cohort (80 patients with *CEBPA*dmbZIP, and 5 patients with *CEBPA*dmnonbZIP). Univariate analysis showed that R/RAML patients with *CEBPA*dmnonbZIP were associated with shorter OS compared to those with *CEBPA*dmbZIP (HR = 2.881, 95% CI = 1.021–8.131, *P* = .046, the Kaplan-Meier curves are shown in Figure 4).

**Figure 5.**
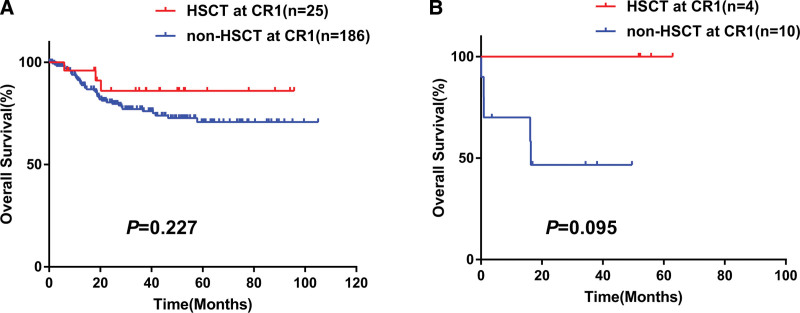
Landmark analysis of CEBPAdmbZIP and CEBPAdmnonbZIP AML (A and B). CEBPAdm = double-mutated *CEBPA*, CEBPAdmnonbZIP = mutations did not occur in bZIP region, HSCT = hematopoietic stem cell transplantation.

To identify the effect of transplantation, we compared OS by landmark analysis in each group according to whether underwent HSCT during CR1. The Kaplan-Meier curves in Figure 5 suggested that HSCT during CR1 did not improve OS in *CEBPA*dmbZIP group, but was associated with a trend to prolong OS in *CEBPA*dmnonbZIP group (*CEBPA*dmbZIP: *P* = .227; *CEBPA*dmnonbZIP: *P* = .095).

## 4. DISCUSSION

In this study, we analyzed 2211 newly-diagnosed AML and identified *CEBPA*dm in 10.8% of the patients. We mainly focus on the heterogeneity of *CEBPA*dm, therefore we performed a detailed analysis of the different types of *CEBPA*dm according to bZIP region mutations. We found that the presence of *CEBPA*dmnonbZIP is associated with a lower prevalence of *GATA2* mutations and poor outcome.

Previous studies showed that *GATA2* mutations occurred in 18% to 41% of patients with *CEBPA*dm AML, which were obviously more common than those of *CEBPA*wt group or *CEBPA*sm group.^15–17^ Similarly, in our *CEBPA*dm cohort, *GATA2* mutations occurred in 28.04% of patients. Studies also found that *CEBPA*dm shared similar biologic features with *CEBPA* bZIP including the high prevalence of *GATA2* mutations.^8,9^ Intriguingly, we found that different incidences of *GATA2* mutations existed between the *CEBPA*dmbZIP group and the *CEBPA*dmnonbZIP group (30.29% vs 0%). This finding suggests that AML bearing *CEBPA*dm with bZIP and without bZIP might have different pathogenesis and be classified as distinctive entities.

Recent studies demonstrated that *CEBPA* mutations involving bZIP region were associated with favorable prognosis in both biallelic and single mutations.^2,8,9^ The favorable category in the new ICC of myeloid neoplasms and acute leukemia^10^ has been updated to *CEBPA* with inframe bZIP mutations instead of biallelic mutations. Simultaneously, the definition has changed to AML with *CEBPA* mutation, which includes biallelic as well as single mutation located in the bZIP region in the 5th edition of WHO Classification of Haematolymphoid Tumours.^11^ The difference between the 2 classifications mentioned above was whether *CEBPA*dmnonbZIP was included. Until now, few studies have been reported about the outcomes of *CEBPA*dmnonbZIP patients. Although we did not find difference in OS and EFS between *CEBPA*dmnonbZIP group and *CEBPA*dmbZIP group, *CEBPA*dmnonbZIP was associated with shorter OS when censored at HSCT during CR1. However, we did not find statistical difference in EFS censored at HSCT during CR1, which prompted us to investigate the outcomes of R/RAML patients. Further analysis showed that R/RAML with *CEBPA*dmnonbZIP were associated with poorer OS compared to those with *CEBPA*dmbZIP. Schlenk et al^18^ revealed that the outcome of relapsed *CEBPA*dm patients could be improved by a high rate of second CR followed by allo-HSCT extending the survival to more than 2 years after relapse. Similarly, the median interval from relapse or refractoriness to death of 27 R/RAML patients with *CEBPA*dmbZIP who underwent allo-HSCT after relapse was 29.67 months in our study. In R/RAML with *CEBPA*dmnonbZIP group, 1 patient underwent allo-HSCT in CR2 and 1 patient received allo-HSCT after 2 induction treatments for NR, the interval from relapse or refractoriness to death was 14.30 and 5.50 months, respectively. Hence, a potential difference between the *CEBPA*dmnonbZIP group and *CEBPA*dmbZIP group was the outcome after relapse and refractoriness, but not in CR1. Thus, these data further suggested that AML with *CEBPA*dmbZIP and *CEBPA*dmnonbZIP should be regarded as distinctive AML entities.

Several limitations existed in our study. This was a retrospective study and the treatment regimens could not be uniform, although the induction chemotherapy regimens were balanced between the 2 groups in our cohort. Another limitation was the limited sample size of patients with *CEBPA*dmnonbZIP. Future larger and prospective studies are needed to clarify the results.

In conclusion, *CEBPA*dmnonbZIP AML was associated with inferior outcome mainly due to shorter survival after refractoriness or relapse compared to *CEBPA*dmbZIP AML, which suggested that they were distinctive entities.

## ACKNOWLEDGMENTS

Funding for this research was provided by the National Key Research and Development Program of China (2021YFC2500300), the National Natural Science Foundation of China(82141122), CAMS Innovation Fund for Medical Sciences (2020-I2M-C&T-B-084), Tianjin Municipal Science and Technology Commission Grant (21ZXGWSY00030), and Haihe Laboratory of Cell Ecosystem Innovation Fund (HH22KYZX0039).

## AUTHOR CONTRIBUTIONS

J.W., Y.M., and H.W. contributed to the study design. Y.H., S.L., J..Z., and B.L. were involved in analyzing and interpreting the data. H.W. and Y.H. wrote the report.

## Supplementary Material


