# Supplementary material for: Heterogeneity analysis of the CEBPAdm AML based on bZIP region mutations
Source: Blood Sci. 2023 Mar 6;5(2):101–5. doi: 10.1097/BS9.0000000000000153 (PMC10205380; doi:10.1097/BS9.0000000000000153)
Supplement: Supplementary file 1 [file bs9-5-101-s001.pdf]

Supplementary Table1. The baseline data of the 239 AML patients with CEBPAdm

**Characteristics**	**n=239**
**Age**,years median (IQR)	40(30,48)
**Sex**	
Male	144
Female	95
**Cytogenetics**	
Normal	184
Abnormal	48
unknown	7
**Induction chemotherapy**	
HAD	76
DA	133
IA	21
Others	9
**CR1-HSCT**	30/239
**OS, months** median (IQR)	33.53(17.20,59.13)
**EFS,months** median (IQR)	23.70(10.30,56.00)

Abbreviations:

HAD: Homoharringtonine, Cytarabine, and Daunorubicin; DA: Daunorubicin and Cytarabine; IA: Idarubicin and Cytarabine; CR1-HSCT: HSCT during the first complete remission phase; OS: Overall survival; EFS: Event-free survival.
